# 1,3-Bis(4-fluoro­phen­yl)-*N*,*N*′-(propane-1,3-diyl­idene)dihydroxyl­amine

**DOI:** 10.1107/S1600536811026547

**Published:** 2011-07-09

**Authors:** S. Samshuddin, Ray J. Butcher, Mehmet Akkurt, B. Narayana, H. S. Yathirajan, B. K. Sarojini

**Affiliations:** a Department of Studies in Chemistry, Mangalore University, Mangalagangotri 574 199, India; b Department of Chemistry, Howard University, 525 College Street NW, Washington, DC 20059, USA; c Department of Physics, Faculty of Sciences, Erciyes University, 38039 Kayseri, Turkey; d Department of Studies in Chemistry, University of Mysore, Manasagangotri, Mysore 570 006, India; e Department of Chemistry, P.A. College of Engineering, Nadupadavu, Mangalore 574 153, India

## Abstract

The title compound, C_15_H_12_F_2_N_2_O_2_, crystallizes with two mol­ecules (*A* and *B*) in the asymmetric unit. Both aromatic rings of both mol­ecules are disordered over two orientations [occupancy ratios of 0.768 (3):0.232 (3) and 0.770 (3):0.230 (3) for mol­ecule *A* and 0.789 (3):0.211 (3) and 0.789 (3):0.211 (3) for mol­ecule *B*]. The dihedral angles between the planes of the major and minor components of the disordered aromatic rings are 72.0 (4) and 71.2 (4)° for mol­ecule *A*, and 70.2 (4) and 71.5 (2)° for mol­ecule *B*. In the crystal, both mol­ecules form inversion dimers with *R*_2_^2^(6) ring motifs *via* pairs of inter­molecular O—H⋯N hydrogen bonds. The dimers are linked, forming zigzag *C*(7) chains along the *c* axis. Weak C—H⋯π inter­actions help to consolidate the packing.

## Related literature

For related 4,4′-difluoro chalcone and oxime structures and background references, see: Baktır *et al.* (2011*a* ▶,*b* ▶); Fun *et al.* (2010*a* ▶,*b* ▶); Jasinski *et al.* (2010*a* ▶,*b* ▶). For hydrogen-bond motifs, see: Bernstein *et al.* (1995 ▶).
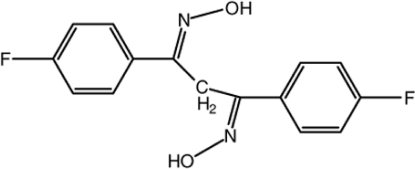


## Experimental

### 

#### Crystal data

C_15_H_12_F_2_N_2_O_2_
*M* *_r_* = 290.27
Triclinic, 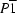

*a* = 9.9233 (7) Å
*b* = 10.4236 (6) Å
*c* = 13.2422 (11) Å
α = 86.419 (6)°
β = 79.205 (7)°
γ = 89.932 (5)°
*V* = 1342.78 (17) Å^3^
*Z* = 4
Cu *K*α radiation
μ = 0.99 mm^−1^
*T* = 295 K
0.42 × 0.28 × 0.25 mm

#### Data collection

Oxford Diffraction Xcalibur Ruby Gemini diffractometer
Absorption correction: multi-scan (*CrysAlis PRO*; Oxford Diffraction, 2007 ▶) *T*_min_ = 0.754, *T*_max_ = 0.782
7806 measured reflections
7849 independent reflections
5008 reflections with *I* > 2σ(*I*)
*R*_int_ = 0.000

#### Refinement

*R*[*F* ^2^ > 2σ(*F* ^2^)] = 0.083
*wR*(*F* ^2^) = 0.236
*S* = 1.01
7849 reflections
376 parameters
H-atom parameters constrained
Δρ_max_ = 0.34 e Å^−3^
Δρ_min_ = −0.22 e Å^−3^

### 

Data collection: *CrysAlis PRO* (Oxford Diffraction, 2007 ▶); cell refinement: *CrysAlis PRO*; data reduction: *CrysAlis RED*; program(s) used to solve structure: *SHELXS97* (Sheldrick, 2008 ▶); program(s) used to refine structure: *SHELXL97* (Sheldrick, 2008 ▶); molecular graphics: *ORTEP-3 for Windows* (Farrugia, 1997 ▶); software used to prepare material for publication: *WinGX* (Farrugia, 1999 ▶) and *PLATON* (Spek, 2009 ▶).

## Supplementary Material

Crystal structure: contains datablock(s) global, I. DOI: 10.1107/S1600536811026547/hb5938sup1.cif

Structure factors: contains datablock(s) I. DOI: 10.1107/S1600536811026547/hb5938Isup2.hkl

Supplementary material file. DOI: 10.1107/S1600536811026547/hb5938Isup3.cml

Additional supplementary materials:  crystallographic information; 3D view; checkCIF report

## Figures and Tables

**Table 1:** Hydrogen-bond geometry (Å, °) *Cg*1, *Cg*4, *Cg*5 and *Cg*8 are the centroids of the C3*A*–C8*A*, C9*C*–C14*C*, C3*B*–C8*B* and C9*D*–C14*D* benzene rings, respectively.

*D*—H⋯*A*	*D*—H	H⋯*A*	*D*⋯*A*	*D*—H⋯*A*
O1*A*—H1*A*⋯N2*B*^i^	0.82	2.06	2.782 (4)	146
O1*B*—H1*B*⋯N2*A*	0.82	2.07	2.778 (4)	145
O2*A*—H2*A*⋯N1*B*	0.82	2.03	2.750 (4)	147
O2*B*—H2*B*⋯N1*A*^ii^	0.82	2.04	2.759 (4)	146
C10*A*—H10*A*⋯*Cg*5^iii^	0.93	2.94	3.690 (3)	139
C13*A*—H13*A*⋯*Cg*5^iv^	0.93	2.98	3.746 (3)	141
C14*B*—H14*C*⋯*Cg*1^iv^	0.93	2.94	3.702 (5)	140
C4*C*—H4*CA*⋯*Cg*8^iii^	0.93	2.92	3.674 (11)	139
C7*C*—H7*CA*⋯*Cg*8^iv^	0.93	2.97	3.749 (11)	143
C4*D*—H4*DA*⋯*Cg*4^iv^	0.93	2.90	3.651 (10)	139

Symmetry codes: (i) 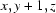; (ii) 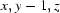; (iii) 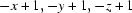; (iv) 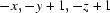.

**Fig. 1.**
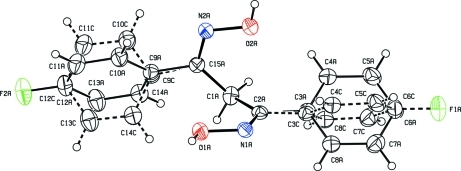
One of the two molecules of (I) in the asymmetric unit, showing displacement ellipsoids for non-H atoms drawn at the 30% probability level.

**Fig. 2.**
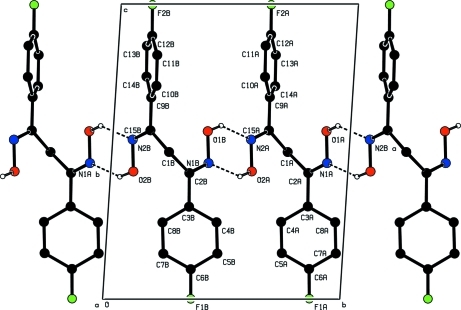
View of inversion dimers of (I) in the unit cell, with R22(6) ring motifs, forming by intermolecular O—H···N hydrogen bonds. Hydrogen atoms not involved in H-bonds have been omitted for clarity.

**Fig. 3.**
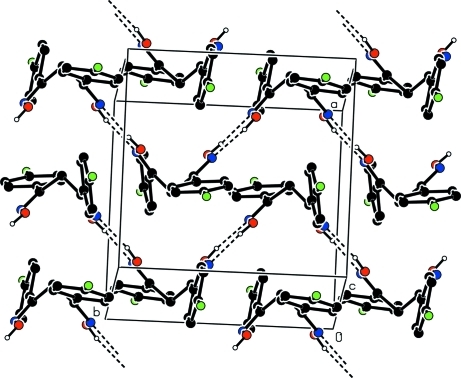
The crystal packing and hydrogen bonding of (I) shown down the c axis. Hydrogen atoms not involved in H-bonds have been omitted for clarity.

## supplementary crystallographic information

### Comment

In continuation of our work on the syntheses and structures of derivatives of 4,4'-difluoro chalcones and oximes (Fun *et al.*, 2010*a*,*b*; Jasinski *et al.*, 2010*a*,*b*; Baktır *et al.*, 2011*a*,*b*), the title compound (I) was prepared and its crystal structure is now reported.

In the title compound (I), two symmetry independent molecules 1 (with F1A) and 2 (with F1B) exist in the asymmetric unit. Fig. 1 shows one of them. The dihedral angles between the planes of the major and minor components of the disordered aromatic rings of (I) are 72.0 (4)°, 71.2 (4)° for molecule 1, and 70.2 (4)°, 71.5 (2)° for molecule 2, respectively. The dihedral angle between the two aromatic rings is 89.09 (17)° (major component), 89.6 (5) ° (minor component) for molecule 1, and 88.7 (2)° (major component), 87.4 (7)° (minor component) for molecule 2, respectively.

In the crystal, intermolecular O—H···N hydrogen bonds (Table 1, Fig. 2) connect molecules, forming inversion dimers with *R*_2_^2^(6) ring motifs (Bernstein *et al.*, 1995). These interactions link the symmetry-related molecules into as infinite zigzag chains along the *c* axis (Fig. 3). In addition, C—H···π interactions are observed (Table 1).

### Experimental

A solution of 2,3-dibromo-1,3-bis(4-fluorophenyl)propan-1-one (4.04 g, 0.01 mol) and hydroxylamine hydrochloride (1.4 g, 0.02 mol) in 25 ml e thanol containing 5 ml of triethylamine was refluxed for 12 h. The reaction mixture was cooled and poured into 50 ml ice-cold water. The precipitate formed was collected by filtration and dried. Colourless prisms of (I) were grown from DMSO by slow evaporation and yield of the compound was 72%. (m.p. 485 K).

### Refinement

H atoms were placed in idealized positions with d(C—H) = 0.97 (CH_2_) and 0.93 Å (CH) and d(O—H) = 0.82 (OH) and refined using a riding model with *U*_iso_(H) fixed at 1.5 *U*_eq_(O) for OH and 1.2 *U*_eq_(C) for CH and CH_2_. The two aromatic rings of two molecules in the asymmetric unit are disordered over two sites with the refined occupancy ratios of 0.768 (3): 0.232 (3) and 0.770 (3): 0.230 (3) for molecule 1 (with F1A), and 0.789 (3): 0.211 (3) and 0.789 (3): 0.211 (3) for molecule 2 (with F1B). Poorly fitted reflections were omitted from the refinement.

### Crystal data

**Table:**

C_15_H_12_F_2_N_2_O_2_	*Z* = 4
*M**_r_* = 290.27	*F*(000) = 600
Triclinic, *P*1	*D*_x_ = 1.436 Mg m^−^^3^
Hall symbol: -P 1	Cu *K*α radiation, λ = 1.54178 Å
*a* = 9.9233 (7) Å	Cell parameters from 1758 reflections
*b* = 10.4236 (6) Å	θ = 4.5–73.4°
*c* = 13.2422 (11) Å	µ = 0.99 mm^−^^1^
α = 86.419 (6)°	*T* = 295 K
β = 79.205 (7)°	Prism, colourless
γ = 89.932 (5)°	0.42 × 0.28 × 0.25 mm
*V* = 1342.78 (17) Å^3^	

### Data collection

**Table:**

Oxford Diffraction Xcalibur Ruby Gemini diffractometer	7849 independent reflections
Radiation source: Enhance (Cu) X-ray Source	5008 reflections with *I* > 2σ(*I*)
graphite	*R*_int_ = 0.000
Detector resolution: 10.5081 pixels mm^-1^	θ_max_ = 67.5°, θ_min_ = 5.3°
ω scans	*h* = −11→11
Absorption correction: multi-scan (*CrysAlis PRO*; Oxford Diffraction, 2007)	*k* = −12→12
*T*_min_ = 0.754, *T*_max_ = 0.782	*l* = −15→15
7806 measured reflections	

### Refinement

**Table:**

Refinement on *F*^2^	Primary atom site location: structure-invariant direct methods
Least-squares matrix: full	Secondary atom site location: difference Fourier map
*R*[*F*^2^ > 2σ(*F*^2^)] = 0.083	Hydrogen site location: inferred from neighbouring sites
*wR*(*F*^2^) = 0.236	H-atom parameters constrained
*S* = 1.01	*w* = 1/[σ^2^(*F*_o_^2^) + (0.0848*P*)^2^ + 1.187*P*] where *P* = (*F*_o_^2^ + 2*F*_c_^2^)/3
7849 reflections	(Δ/σ)_max_ < 0.001
376 parameters	Δρ_max_ = 0.34 e Å^−^^3^
0 restraints	Δρ_min_ = −0.22 e Å^−^^3^

### Special details

**Table:**

Geometry. Bond distances, angles *etc*. have been calculated using the rounded fractional coordinates. All su's are estimated from the variances of the (full) variance-covariance matrix. The cell e.s.d.'s are taken into account in the estimation of distances, angles and torsion angles
Refinement. Refinement on *F*^2^ for ALL reflections except those flagged by the user for potential systematic errors. Weighted *R*-factors *wR* and all goodnesses of fit *S* are based on *F*^2^, conventional *R*-factors *R* are based on *F*, with *F* set to zero for negative *F*^2^. The observed criterion of *F*^2^ > σ(*F*^2^) is used only for calculating -*R*-factor-obs *etc*. and is not relevant to the choice of reflections for refinement. *R*-factors based on *F*^2^ are statistically about twice as large as those based on *F*, and *R*-factors based on ALL data will be even larger.

### Fractional atomic coordinates and isotropic or equivalent isotropic displacement parameters (Å^2^)

**Table:**

	*x*	*y*	*z*	*U*_iso_*/*U*_eq_	Occ. (<1)
F1A	0.0906 (3)	0.8715 (3)	0.00153 (16)	0.0858 (10)	
F2A	−0.1596 (3)	0.6327 (3)	0.99701 (16)	0.0859 (10)	
O1A	0.1328 (3)	0.8991 (2)	0.56874 (16)	0.0516 (8)	
O2A	0.1813 (3)	0.5948 (2)	0.43283 (17)	0.0541 (8)	
N1A	0.1482 (3)	0.9103 (2)	0.46093 (19)	0.0422 (8)	
N2A	0.1393 (3)	0.5835 (2)	0.54025 (19)	0.0456 (8)	
C1A	−0.0274 (3)	0.7411 (3)	0.4983 (2)	0.0465 (10)	
C2A	0.0698 (3)	0.8334 (3)	0.4274 (2)	0.0388 (9)	
C3A	0.0740 (3)	0.8406 (3)	0.31399 (16)	0.0403 (10)	0.768 (3)
C4A	0.0563 (4)	0.7322 (2)	0.2616 (2)	0.0572 (16)	0.768 (3)
C5A	0.0615 (4)	0.7433 (2)	0.1558 (2)	0.0603 (17)	0.768 (3)
C6A	0.0844 (4)	0.8627 (3)	0.10236 (16)	0.0566 (11)	0.768 (3)
C7A	0.1021 (4)	0.9711 (2)	0.1548 (2)	0.0615 (17)	0.768 (3)
C8A	0.0969 (3)	0.9600 (2)	0.2606 (2)	0.0490 (12)	0.768 (3)
C9A	−0.0120 (3)	0.6510 (3)	0.68451 (15)	0.0409 (10)	0.770 (3)
C10A	0.0764 (2)	0.6255 (3)	0.7530 (2)	0.0552 (14)	0.770 (3)
C11A	0.0263 (3)	0.6198 (4)	0.85855 (19)	0.0633 (17)	0.770 (3)
C12A	−0.1122 (3)	0.6396 (4)	0.89551 (16)	0.0575 (11)	0.770 (3)
C13A	−0.2007 (2)	0.6651 (4)	0.8270 (2)	0.0639 (19)	0.770 (3)
C14A	−0.1505 (3)	0.6708 (3)	0.7215 (2)	0.0502 (14)	0.770 (3)
C15A	0.0390 (3)	0.6565 (3)	0.5715 (2)	0.0402 (9)	
C6C	0.0856 (11)	0.8607 (12)	0.1053 (5)	0.0566 (11)	0.232 (3)
C7C	−0.0382 (9)	0.8401 (10)	0.1736 (7)	0.0615 (17)	0.232 (3)
C8C	−0.0414 (8)	0.8352 (10)	0.2791 (7)	0.0490 (12)	0.232 (3)
C9C	−0.0278 (11)	0.6568 (10)	0.6836 (5)	0.0409 (10)	0.230 (3)
C10C	−0.0324 (11)	0.5375 (8)	0.7374 (7)	0.0552 (14)	0.230 (3)
C11C	−0.0760 (12)	0.5278 (9)	0.8437 (7)	0.0633 (17)	0.230 (3)
C12C	−0.1149 (13)	0.6373 (11)	0.8962 (5)	0.0575 (11)	0.230 (3)
C13C	−0.1102 (12)	0.7565 (9)	0.8424 (7)	0.0639 (19)	0.230 (3)
C14C	−0.0666 (11)	0.7663 (8)	0.7361 (7)	0.0502 (14)	0.230 (3)
C3C	0.0791 (10)	0.8508 (11)	0.3164 (5)	0.0403 (10)	0.232 (3)
C4C	0.2028 (8)	0.8714 (10)	0.2482 (7)	0.0572 (16)	0.232 (3)
C5C	0.2061 (9)	0.8764 (11)	0.1426 (6)	0.0603 (17)	0.232 (3)
F1B	0.6590 (3)	0.3717 (3)	0.00105 (16)	0.0917 (10)	
F2B	0.4100 (3)	0.1317 (3)	0.99706 (17)	0.0914 (10)	
O1B	0.3328 (3)	0.3986 (2)	0.56877 (17)	0.0530 (8)	
O2B	0.3525 (3)	0.0955 (2)	0.43261 (17)	0.0542 (8)	
N1B	0.3716 (3)	0.4104 (2)	0.46091 (18)	0.0427 (8)	
N2B	0.3404 (3)	0.0842 (2)	0.54041 (19)	0.0446 (8)	
C1B	0.5275 (3)	0.2410 (3)	0.4984 (3)	0.0486 (10)	
C2B	0.4666 (3)	0.3322 (3)	0.4272 (2)	0.0418 (9)	
C3B	0.5192 (3)	0.3400 (3)	0.31310 (16)	0.0414 (10)	0.789 (3)
C4B	0.5230 (3)	0.4599 (2)	0.2604 (2)	0.0518 (12)	0.789 (3)
C5B	0.5702 (4)	0.4718 (2)	0.1545 (2)	0.0632 (17)	0.789 (3)
C6B	0.6136 (4)	0.3638 (3)	0.10139 (16)	0.0615 (13)	0.789 (3)
C7B	0.6098 (4)	0.2439 (3)	0.1541 (2)	0.0631 (17)	0.789 (3)
C8B	0.5626 (3)	0.2320 (2)	0.2600 (2)	0.0545 (14)	0.789 (3)
C9B	0.4219 (6)	0.1513 (5)	0.6833 (3)	0.0426 (10)	0.789 (3)
C10B	0.5395 (4)	0.1700 (4)	0.7227 (3)	0.0524 (14)	0.789 (3)
C11B	0.5368 (5)	0.1652 (5)	0.8271 (4)	0.0658 (19)	0.789 (3)
C12B	0.4134 (8)	0.1387 (6)	0.8921 (4)	0.0603 (15)	0.789 (3)
C13B	0.2955 (5)	0.1192 (5)	0.8581 (4)	0.0659 (17)	0.789 (3)
C14B	0.2991 (5)	0.1256 (5)	0.7540 (3)	0.0554 (14)	0.789 (3)
C15B	0.4257 (3)	0.1567 (3)	0.5718 (2)	0.0427 (10)	
C6D	0.6141 (12)	0.3597 (14)	0.1079 (6)	0.0615 (13)	0.211 (3)
C7D	0.7021 (9)	0.3343 (12)	0.1768 (8)	0.0631 (17)	0.211 (3)
C8D	0.6514 (10)	0.3284 (11)	0.2822 (8)	0.0545 (14)	0.211 (3)
C9D	0.4275 (18)	0.1589 (12)	0.6897 (7)	0.0426 (10)	0.211 (3)
C10D	0.4407 (14)	0.2673 (10)	0.7432 (9)	0.0524 (14)	0.211 (3)
C11D	0.4343 (16)	0.2554 (11)	0.8492 (9)	0.0658 (19)	0.211 (3)
C12D	0.415 (2)	0.1352 (13)	0.9016 (7)	0.0603 (15)	0.211 (3)
C13D	0.4016 (16)	0.0267 (11)	0.8480 (9)	0.0659 (17)	0.211 (3)
C14D	0.4080 (15)	0.0386 (11)	0.7421 (9)	0.0554 (14)	0.211 (3)
C3D	0.5127 (11)	0.3479 (12)	0.3188 (6)	0.0414 (10)	0.211 (3)
C4D	0.4247 (8)	0.3733 (11)	0.2499 (8)	0.0518 (12)	0.211 (3)
C5D	0.4754 (11)	0.3792 (12)	0.1445 (7)	0.0632 (17)	0.211 (3)
H11A	0.08550	0.60280	0.90440	0.0760*	0.770 (3)
H13A	−0.29330	0.67830	0.85170	0.0770*	0.770 (3)
H14A	−0.20970	0.68780	0.67560	0.0600*	0.770 (3)
H1AA	−0.09910	0.79020	0.53830	0.0560*	
H1A	0.18920	0.94580	0.58680	0.0770*	
H1AB	−0.07070	0.68660	0.45660	0.0560*	
H2A	0.24540	0.54620	0.41570	0.0810*	
H4AA	0.04100	0.65230	0.29730	0.0690*	0.768 (3)
H5AA	0.04970	0.67080	0.12070	0.0720*	0.768 (3)
H7AA	0.11740	1.05100	0.11900	0.0740*	0.768 (3)
H8AA	0.10870	1.03250	0.29560	0.0590*	0.768 (3)
H10A	0.16910	0.61230	0.72830	0.0660*	0.770 (3)
H4CA	0.28340	0.88190	0.27310	0.0690*	0.232 (3)
H5CA	0.28890	0.89020	0.09690	0.0720*	0.232 (3)
H7CA	−0.11880	0.82970	0.14860	0.0740*	0.232 (3)
H8CA	−0.12420	0.82140	0.32480	0.0590*	0.232 (3)
H10B	−0.00640	0.46430	0.70230	0.0660*	0.230 (3)
H11B	−0.07910	0.44800	0.87970	0.0760*	0.230 (3)
H13B	−0.13620	0.82980	0.87750	0.0770*	0.230 (3)
H14B	−0.06350	0.84610	0.70010	0.0600*	0.230 (3)
H14C	0.21850	0.11280	0.72950	0.0670*	0.789 (3)
H7BA	0.63890	0.17170	0.11860	0.0760*	0.789 (3)
H8BA	0.56010	0.15180	0.29520	0.0650*	0.789 (3)
H1B	0.27560	0.45320	0.58760	0.0790*	
H2B	0.30480	0.04020	0.41470	0.0810*	
H10C	0.62230	0.18610	0.67760	0.0630*	0.789 (3)
H1BA	0.57880	0.29060	0.53820	0.0580*	
H11C	0.61600	0.17950	0.85300	0.0790*	0.789 (3)
H1BB	0.59200	0.18630	0.45710	0.0580*	
H4BA	0.49390	0.53220	0.29590	0.0620*	0.789 (3)
H13C	0.21370	0.10180	0.90420	0.0790*	0.789 (3)
H5BA	0.57270	0.55200	0.11920	0.0760*	0.789 (3)
H10D	0.45380	0.34780	0.70820	0.0630*	0.211 (3)
H11D	0.44310	0.32800	0.88500	0.0790*	0.211 (3)
H13D	0.38850	−0.05370	0.88310	0.0790*	0.211 (3)
H14D	0.39920	−0.03400	0.70630	0.0670*	0.211 (3)
H4DA	0.33190	0.38630	0.27430	0.0620*	0.211 (3)
H5DA	0.41650	0.39610	0.09840	0.0760*	0.211 (3)
H7DA	0.79490	0.32120	0.15240	0.0760*	0.211 (3)
H8DA	0.71030	0.31140	0.32830	0.0650*	0.211 (3)

### Atomic displacement parameters (Å^2^)

**Table:**

	*U*^11^	*U*^22^	*U*^33^	*U*^12^	*U*^13^	*U*^23^
F1A	0.112 (2)	0.105 (2)	0.0427 (12)	0.0046 (16)	−0.0206 (12)	−0.0054 (12)
F2A	0.0852 (18)	0.126 (2)	0.0419 (12)	−0.0055 (15)	0.0004 (11)	−0.0060 (13)
O1A	0.0632 (16)	0.0500 (14)	0.0436 (12)	−0.0012 (11)	−0.0134 (10)	−0.0080 (10)
O2A	0.0627 (16)	0.0546 (14)	0.0423 (12)	0.0091 (11)	−0.0019 (10)	−0.0055 (10)
N1A	0.0432 (14)	0.0431 (14)	0.0407 (14)	0.0033 (11)	−0.0081 (11)	−0.0060 (11)
N2A	0.0565 (16)	0.0406 (14)	0.0388 (14)	0.0019 (12)	−0.0062 (11)	−0.0047 (11)
C1A	0.0358 (16)	0.0598 (19)	0.0435 (16)	−0.0011 (14)	−0.0075 (13)	0.0000 (14)
C2A	0.0360 (15)	0.0371 (15)	0.0446 (16)	0.0077 (12)	−0.0096 (12)	−0.0059 (13)
C3A	0.0380 (17)	0.0396 (17)	0.0443 (17)	0.0036 (13)	−0.0100 (13)	−0.0044 (13)
C4A	0.077 (3)	0.044 (2)	0.052 (3)	0.006 (2)	−0.015 (2)	−0.0047 (19)
C5A	0.072 (3)	0.058 (3)	0.058 (3)	0.008 (2)	−0.025 (2)	−0.020 (2)
C6A	0.062 (2)	0.069 (2)	0.0391 (18)	0.0091 (18)	−0.0106 (15)	−0.0036 (16)
C7A	0.072 (3)	0.053 (3)	0.059 (3)	−0.002 (2)	−0.014 (2)	0.005 (2)
C8A	0.048 (2)	0.047 (2)	0.052 (2)	0.0004 (17)	−0.0096 (18)	−0.0035 (18)
C9A	0.0369 (18)	0.0445 (17)	0.0416 (17)	0.0039 (13)	−0.0084 (13)	−0.0016 (13)
C10A	0.048 (2)	0.071 (3)	0.048 (2)	0.010 (2)	−0.0121 (19)	−0.006 (2)
C11A	0.065 (3)	0.074 (3)	0.053 (3)	0.003 (2)	−0.019 (2)	0.003 (2)
C12A	0.064 (2)	0.069 (2)	0.0378 (17)	−0.0094 (18)	−0.0046 (15)	−0.0049 (16)
C13A	0.049 (3)	0.088 (4)	0.052 (3)	−0.002 (2)	−0.001 (2)	−0.010 (2)
C14A	0.038 (2)	0.062 (3)	0.051 (2)	0.0020 (18)	−0.0089 (17)	−0.0056 (19)
C15A	0.0400 (16)	0.0392 (16)	0.0417 (16)	−0.0016 (12)	−0.0088 (12)	−0.0016 (13)
C6C	0.062 (2)	0.069 (2)	0.0391 (18)	0.0091 (18)	−0.0106 (15)	−0.0036 (16)
C7C	0.072 (3)	0.053 (3)	0.059 (3)	−0.002 (2)	−0.014 (2)	0.005 (2)
C8C	0.048 (2)	0.047 (2)	0.052 (2)	0.0004 (17)	−0.0096 (18)	−0.0035 (18)
C9C	0.0369 (18)	0.0445 (17)	0.0416 (17)	0.0039 (13)	−0.0084 (13)	−0.0016 (13)
C10C	0.048 (2)	0.071 (3)	0.048 (2)	0.010 (2)	−0.0121 (19)	−0.006 (2)
C11C	0.065 (3)	0.074 (3)	0.053 (3)	0.003 (2)	−0.019 (2)	0.003 (2)
C12C	0.064 (2)	0.069 (2)	0.0378 (17)	−0.0094 (18)	−0.0046 (15)	−0.0049 (16)
C13C	0.049 (3)	0.088 (4)	0.052 (3)	−0.002 (2)	−0.001 (2)	−0.010 (2)
C14C	0.038 (2)	0.062 (3)	0.051 (2)	0.0020 (18)	−0.0089 (17)	−0.0056 (19)
C3C	0.0380 (17)	0.0396 (17)	0.0443 (17)	0.0036 (13)	−0.0100 (13)	−0.0044 (13)
C4C	0.077 (3)	0.044 (2)	0.052 (3)	0.006 (2)	−0.015 (2)	−0.0047 (19)
C5C	0.072 (3)	0.058 (3)	0.058 (3)	0.008 (2)	−0.025 (2)	−0.020 (2)
F1B	0.111 (2)	0.112 (2)	0.0441 (13)	−0.0035 (16)	0.0064 (12)	−0.0061 (13)
F2B	0.100 (2)	0.133 (2)	0.0439 (13)	0.0124 (17)	−0.0194 (12)	−0.0090 (14)
O1B	0.0651 (16)	0.0505 (14)	0.0426 (12)	0.0085 (11)	−0.0069 (10)	−0.0075 (10)
O2B	0.0622 (16)	0.0559 (14)	0.0456 (13)	−0.0048 (11)	−0.0122 (11)	−0.0063 (10)
N1B	0.0476 (15)	0.0419 (14)	0.0378 (13)	0.0034 (11)	−0.0051 (11)	−0.0056 (10)
N2B	0.0524 (16)	0.0418 (14)	0.0407 (14)	0.0013 (11)	−0.0105 (11)	−0.0062 (11)
C1B	0.0352 (16)	0.063 (2)	0.0485 (17)	0.0033 (14)	−0.0116 (13)	−0.0004 (15)
C2B	0.0372 (16)	0.0411 (16)	0.0482 (17)	−0.0056 (12)	−0.0109 (13)	−0.0035 (13)
C3B	0.0356 (16)	0.0428 (17)	0.0462 (17)	−0.0016 (13)	−0.0080 (13)	−0.0059 (14)
C4B	0.050 (2)	0.049 (2)	0.057 (2)	0.0044 (18)	−0.0127 (19)	−0.0013 (19)
C5B	0.066 (3)	0.064 (3)	0.058 (3)	−0.002 (2)	−0.011 (2)	0.007 (2)
C6B	0.065 (2)	0.078 (3)	0.0380 (18)	−0.0030 (19)	−0.0003 (16)	−0.0041 (18)
C7B	0.060 (3)	0.062 (3)	0.064 (3)	0.002 (2)	0.003 (2)	−0.024 (2)
C8B	0.058 (3)	0.049 (2)	0.054 (2)	0.0013 (19)	−0.003 (2)	−0.0060 (19)
C9B	0.0388 (18)	0.0447 (17)	0.0456 (18)	0.0051 (14)	−0.0108 (13)	−0.0047 (14)
C10B	0.039 (2)	0.067 (3)	0.054 (2)	0.0043 (18)	−0.0140 (17)	−0.010 (2)
C11B	0.062 (3)	0.084 (4)	0.057 (3)	0.011 (2)	−0.022 (2)	−0.015 (2)
C12B	0.072 (3)	0.072 (3)	0.0400 (19)	0.0120 (19)	−0.0177 (18)	−0.0063 (18)
C13B	0.055 (3)	0.081 (3)	0.057 (3)	−0.007 (2)	0.001 (2)	−0.003 (2)
C14B	0.046 (2)	0.069 (3)	0.052 (2)	−0.0058 (19)	−0.0110 (19)	−0.005 (2)
C15B	0.0385 (16)	0.0447 (17)	0.0452 (17)	0.0051 (13)	−0.0085 (13)	−0.0030 (13)
C6D	0.065 (2)	0.078 (3)	0.0380 (18)	−0.0030 (19)	−0.0003 (16)	−0.0041 (18)
C7D	0.060 (3)	0.062 (3)	0.064 (3)	0.002 (2)	0.003 (2)	−0.024 (2)
C8D	0.058 (3)	0.049 (2)	0.054 (2)	0.0013 (19)	−0.003 (2)	−0.0060 (19)
C9D	0.0388 (18)	0.0447 (17)	0.0456 (18)	0.0051 (14)	−0.0108 (13)	−0.0047 (14)
C10D	0.039 (2)	0.067 (3)	0.054 (2)	0.0043 (18)	−0.0140 (17)	−0.010 (2)
C11D	0.062 (3)	0.084 (4)	0.057 (3)	0.011 (2)	−0.022 (2)	−0.015 (2)
C12D	0.072 (3)	0.072 (3)	0.0400 (19)	0.0120 (19)	−0.0177 (18)	−0.0063 (18)
C13D	0.055 (3)	0.081 (3)	0.057 (3)	−0.007 (2)	0.001 (2)	−0.003 (2)
C14D	0.046 (2)	0.069 (3)	0.052 (2)	−0.0058 (19)	−0.0110 (19)	−0.005 (2)
C3D	0.0356 (16)	0.0428 (17)	0.0462 (17)	−0.0016 (13)	−0.0080 (13)	−0.0059 (14)
C4D	0.050 (2)	0.049 (2)	0.057 (2)	0.0044 (18)	−0.0127 (19)	−0.0013 (19)
C5D	0.066 (3)	0.064 (3)	0.058 (3)	−0.002 (2)	−0.011 (2)	0.007 (2)

### Geometric parameters (Å, °)

**Table:**

F1A—C6A	1.323 (3)	C8A—H8AA	0.9300
F1A—C6C	1.363 (7)	C8C—H8CA	0.9300
F2A—C12A	1.336 (3)	C10A—H10A	0.9300
F2A—C12C	1.323 (7)	C10C—H10B	0.9300
F1B—C6B	1.318 (3)	C11A—H11A	0.9300
F1B—C6D	1.399 (8)	C11C—H11B	0.9300
F2B—C12B	1.381 (6)	C13A—H13A	0.9300
F2B—C12D	1.254 (10)	C13C—H13B	0.9300
O1A—N1A	1.405 (3)	C14A—H14A	0.9300
O2A—N2A	1.403 (3)	C14C—H14B	0.9300
O1A—H1A	0.8200	C1B—C2B	1.503 (4)
O2A—H2A	0.8200	C1B—C15B	1.505 (4)
O1B—N1B	1.406 (3)	C2B—C3D	1.422 (8)
O2B—N2B	1.407 (3)	C2B—C3B	1.500 (3)
O1B—H1B	0.8200	C3B—C4B	1.390 (4)
O2B—H2B	0.8200	C3B—C8B	1.390 (4)
N1A—C2A	1.273 (4)	C3D—C4D	1.390 (14)
N2A—C15A	1.274 (4)	C3D—C8D	1.390 (15)
N1B—C2B	1.280 (4)	C4B—C5B	1.391 (4)
N2B—C15B	1.278 (4)	C4D—C5D	1.390 (14)
C1A—C2A	1.513 (4)	C5B—C6B	1.390 (4)
C1A—C15A	1.511 (4)	C5D—C6D	1.390 (16)
C2A—C3C	1.455 (7)	C6B—C7B	1.390 (4)
C2A—C3A	1.492 (3)	C6D—C7D	1.390 (14)
C3A—C4A	1.390 (4)	C7B—C8B	1.391 (4)
C3A—C8A	1.390 (4)	C7D—C8D	1.390 (15)
C3C—C4C	1.389 (12)	C9B—C10B	1.383 (7)
C3C—C8C	1.390 (13)	C9B—C14B	1.406 (7)
C4A—C5A	1.390 (4)	C9B—C15B	1.468 (5)
C4C—C5C	1.390 (12)	C9D—C15B	1.566 (10)
C5A—C6A	1.390 (4)	C9D—C10D	1.390 (16)
C5C—C6C	1.390 (14)	C9D—C14D	1.390 (17)
C6A—C7A	1.391 (4)	C10B—C11B	1.376 (6)
C6C—C7C	1.391 (13)	C10D—C11D	1.391 (17)
C7A—C8A	1.390 (4)	C11B—C12B	1.376 (9)
C7C—C8C	1.389 (13)	C11D—C12D	1.389 (17)
C9A—C10A	1.389 (4)	C12B—C13B	1.350 (9)
C9A—C15A	1.485 (3)	C12D—C13D	1.392 (17)
C9A—C14A	1.390 (4)	C13B—C14B	1.370 (7)
C9C—C14C	1.390 (13)	C13D—C14D	1.390 (17)
C9C—C15A	1.508 (7)	C1B—H1BA	0.9700
C9C—C10C	1.390 (13)	C1B—H1BB	0.9700
C10A—C11A	1.391 (4)	C4B—H4BA	0.9300
C10C—C11C	1.390 (13)	C4D—H4DA	0.9300
C11A—C12A	1.390 (4)	C5B—H5BA	0.9300
C11C—C12C	1.390 (14)	C5D—H5DA	0.9300
C12A—C13A	1.390 (4)	C7B—H7BA	0.9300
C12C—C13C	1.390 (14)	C7D—H7DA	0.9300
C13A—C14A	1.390 (4)	C8B—H8BA	0.9300
C13C—C14C	1.390 (13)	C8D—H8DA	0.9300
C1A—H1AA	0.9700	C10B—H10C	0.9300
C1A—H1AB	0.9700	C10D—H10D	0.9300
C4A—H4AA	0.9300	C11B—H11C	0.9300
C4C—H4CA	0.9300	C11D—H11D	0.9300
C5A—H5AA	0.9300	C13B—H13C	0.9300
C5C—H5CA	0.9300	C13D—H13D	0.9300
C7A—H7AA	0.9300	C14B—H14C	0.9300
C7C—H7CA	0.9300	C14D—H14D	0.9300
			
N1A—O1A—H1A	109.00	C13A—C14A—H14A	120.00
N2A—O2A—H2A	110.00	C9A—C14A—H14A	120.00
N1B—O1B—H1B	109.00	C13C—C14C—H14B	120.00
N2B—O2B—H2B	109.00	C9C—C14C—H14B	120.00
O1A—N1A—C2A	112.5 (2)	C2B—C1B—C15B	115.2 (3)
O2A—N2A—C15A	112.4 (2)	N1B—C2B—C1B	121.9 (3)
O1B—N1B—C2B	112.4 (2)	C1B—C2B—C3B	121.3 (3)
O2B—N2B—C15B	112.4 (2)	N1B—C2B—C3B	116.7 (3)
C2A—C1A—C15A	114.7 (3)	N1B—C2B—C3D	113.1 (5)
C1A—C2A—C3A	120.6 (3)	C1B—C2B—C3D	124.8 (5)
C1A—C2A—C3C	125.0 (5)	C4B—C3B—C8B	120.0 (2)
N1A—C2A—C3C	112.6 (5)	C2B—C3B—C8B	122.1 (3)
N1A—C2A—C3A	117.0 (3)	C2B—C3B—C4B	117.9 (3)
N1A—C2A—C1A	122.3 (2)	C2B—C3D—C4D	123.0 (9)
C4A—C3A—C8A	120.0 (2)	C2B—C3D—C8D	116.9 (8)
C2A—C3A—C8A	118.1 (3)	C4D—C3D—C8D	120.0 (8)
C2A—C3A—C4A	121.9 (3)	C3B—C4B—C5B	120.0 (2)
C2A—C3C—C8C	116.9 (7)	C3D—C4D—C5D	120.0 (9)
C4C—C3C—C8C	120.0 (7)	C4B—C5B—C6B	120.0 (2)
C2A—C3C—C4C	122.9 (8)	C4D—C5D—C6D	120.0 (9)
C3A—C4A—C5A	120.0 (2)	F1B—C6B—C5B	121.6 (3)
C3C—C4C—C5C	120.0 (8)	F1B—C6B—C7B	118.4 (3)
C4A—C5A—C6A	120.0 (2)	C5B—C6B—C7B	120.0 (2)
C4C—C5C—C6C	120.0 (8)	F1B—C6D—C7D	123.0 (9)
F1A—C6A—C5A	119.3 (3)	F1B—C6D—C5D	117.0 (8)
F1A—C6A—C7A	120.7 (3)	C5D—C6D—C7D	120.0 (8)
C5A—C6A—C7A	120.0 (2)	C6B—C7B—C8B	120.0 (3)
C5C—C6C—C7C	120.0 (7)	C6D—C7D—C8D	120.0 (9)
F1A—C6C—C7C	121.1 (9)	C3B—C8B—C7B	120.0 (2)
F1A—C6C—C5C	118.9 (8)	C3D—C8D—C7D	120.0 (9)
C6A—C7A—C8A	120.0 (2)	C14B—C9B—C15B	121.3 (5)
C6C—C7C—C8C	120.0 (8)	C10B—C9B—C14B	117.5 (4)
C3A—C8A—C7A	120.0 (2)	C10B—C9B—C15B	121.2 (4)
C3C—C8C—C7C	120.0 (8)	C14D—C9D—C15B	113.7 (9)
C10A—C9A—C14A	120.0 (2)	C10D—C9D—C15B	126.2 (9)
C10A—C9A—C15A	120.9 (3)	C10D—C9D—C14D	120.0 (9)
C14A—C9A—C15A	119.1 (2)	C9B—C10B—C11B	121.5 (4)
C10C—C9C—C15A	114.5 (8)	C9D—C10D—C11D	120.0 (10)
C10C—C9C—C14C	120.0 (7)	C10B—C11B—C12B	118.0 (5)
C14C—C9C—C15A	125.0 (8)	C10D—C11D—C12D	120.1 (10)
C9A—C10A—C11A	120.0 (2)	C11B—C12B—C13B	123.1 (5)
C9C—C10C—C11C	120.0 (8)	F2B—C12B—C11B	118.5 (6)
C10A—C11A—C12A	120.0 (2)	F2B—C12B—C13B	118.5 (6)
C10C—C11C—C12C	120.0 (8)	F2B—C12D—C13D	123.5 (11)
C11A—C12A—C13A	120.0 (2)	F2B—C12D—C11D	116.6 (11)
F2A—C12A—C11A	119.6 (3)	C11D—C12D—C13D	120.0 (10)
F2A—C12A—C13A	120.4 (3)	C12B—C13B—C14B	118.4 (5)
F2A—C12C—C11C	122.3 (9)	C12D—C13D—C14D	120.0 (10)
F2A—C12C—C13C	117.7 (9)	C9B—C14B—C13B	121.4 (5)
C11C—C12C—C13C	120.0 (7)	C9D—C14D—C13D	120.0 (11)
C12A—C13A—C14A	119.9 (2)	N2B—C15B—C1B	122.0 (3)
C12C—C13C—C14C	120.0 (8)	N2B—C15B—C9B	116.6 (3)
C9A—C14A—C13A	120.1 (2)	N2B—C15B—C9D	120.1 (6)
C9C—C14C—C13C	120.0 (8)	C1B—C15B—C9B	121.4 (3)
N2A—C15A—C1A	122.4 (2)	C1B—C15B—C9D	117.9 (6)
N2A—C15A—C9C	122.0 (5)	C2B—C1B—H1BA	108.00
N2A—C15A—C9A	116.0 (3)	C2B—C1B—H1BB	108.00
C1A—C15A—C9A	121.6 (3)	C15B—C1B—H1BA	108.00
C1A—C15A—C9C	115.5 (5)	C15B—C1B—H1BB	108.00
C2A—C1A—H1AB	109.00	H1BA—C1B—H1BB	107.00
C15A—C1A—H1AA	109.00	C3B—C4B—H4BA	120.00
C15A—C1A—H1AB	109.00	C5B—C4B—H4BA	120.00
H1AA—C1A—H1AB	107.00	C5D—C4D—H4DA	120.00
C2A—C1A—H1AA	109.00	C3D—C4D—H4DA	120.00
C5A—C4A—H4AA	120.00	C4B—C5B—H5BA	120.00
C3A—C4A—H4AA	120.00	C6B—C5B—H5BA	120.00
C3C—C4C—H4CA	120.00	C4D—C5D—H5DA	120.00
C5C—C4C—H4CA	120.00	C6D—C5D—H5DA	120.00
C4A—C5A—H5AA	120.00	C8B—C7B—H7BA	120.00
C6A—C5A—H5AA	120.00	C6B—C7B—H7BA	120.00
C6C—C5C—H5CA	120.00	C6D—C7D—H7DA	120.00
C4C—C5C—H5CA	120.00	C8D—C7D—H7DA	120.00
C8A—C7A—H7AA	120.00	C3B—C8B—H8BA	120.00
C6A—C7A—H7AA	120.00	C7B—C8B—H8BA	120.00
C8C—C7C—H7CA	120.00	C7D—C8D—H8DA	120.00
C6C—C7C—H7CA	120.00	C3D—C8D—H8DA	120.00
C3A—C8A—H8AA	120.00	C11B—C10B—H10C	119.00
C7A—C8A—H8AA	120.00	C9B—C10B—H10C	119.00
C3C—C8C—H8CA	120.00	C9D—C10D—H10D	120.00
C7C—C8C—H8CA	120.00	C11D—C10D—H10D	120.00
C9A—C10A—H10A	120.00	C10B—C11B—H11C	121.00
C11A—C10A—H10A	120.00	C12B—C11B—H11C	121.00
C11C—C10C—H10B	120.00	C12D—C11D—H11D	120.00
C9C—C10C—H10B	120.00	C10D—C11D—H11D	120.00
C12A—C11A—H11A	120.00	C12B—C13B—H13C	121.00
C10A—C11A—H11A	120.00	C14B—C13B—H13C	121.00
C10C—C11C—H11B	120.00	C12D—C13D—H13D	120.00
C12C—C11C—H11B	120.00	C14D—C13D—H13D	120.00
C14A—C13A—H13A	120.00	C13B—C14B—H14C	119.00
C12A—C13A—H13A	120.00	C9B—C14B—H14C	119.00
C12C—C13C—H13B	120.00	C9D—C14D—H14D	120.00
C14C—C13C—H13B	120.00	C13D—C14D—H14D	120.00
			
O1A—N1A—C2A—C1A	−0.3 (4)	C10A—C11A—C12A—C13A	0.0 (6)
O1A—N1A—C2A—C3A	177.8 (2)	C11A—C12A—C13A—C14A	0.0 (6)
O2A—N2A—C15A—C1A	1.9 (4)	F2A—C12A—C13A—C14A	−179.4 (4)
O2A—N2A—C15A—C9A	−179.8 (2)	C12A—C13A—C14A—C9A	0.0 (5)
O1B—N1B—C2B—C1B	−0.7 (4)	C15B—C1B—C2B—N1B	53.8 (4)
O1B—N1B—C2B—C3B	−177.9 (2)	C15B—C1B—C2B—C3B	−129.0 (3)
O2B—N2B—C15B—C1B	−1.1 (4)	C2B—C1B—C15B—N2B	55.5 (4)
O2B—N2B—C15B—C9B	−179.6 (3)	C2B—C1B—C15B—C9B	−126.1 (4)
C15A—C1A—C2A—N1A	−53.2 (4)	N1B—C2B—C3B—C4B	33.5 (4)
C2A—C1A—C15A—C9A	125.3 (3)	N1B—C2B—C3B—C8B	−146.1 (3)
C15A—C1A—C2A—C3A	128.9 (3)	C1B—C2B—C3B—C4B	−143.8 (3)
C2A—C1A—C15A—N2A	−56.5 (4)	C1B—C2B—C3B—C8B	36.6 (4)
C1A—C2A—C3A—C4A	−36.0 (4)	C2B—C3B—C4B—C5B	−179.6 (3)
N1A—C2A—C3A—C8A	−33.8 (4)	C8B—C3B—C4B—C5B	0.0 (5)
C1A—C2A—C3A—C8A	144.3 (3)	C2B—C3B—C8B—C7B	179.6 (3)
N1A—C2A—C3A—C4A	145.9 (3)	C4B—C3B—C8B—C7B	0.0 (5)
C2A—C3A—C4A—C5A	−179.8 (3)	C3B—C4B—C5B—C6B	0.0 (5)
C8A—C3A—C4A—C5A	0.0 (5)	C4B—C5B—C6B—F1B	−180.0 (3)
C2A—C3A—C8A—C7A	179.8 (3)	C4B—C5B—C6B—C7B	0.0 (6)
C4A—C3A—C8A—C7A	0.0 (5)	F1B—C6B—C7B—C8B	180.0 (3)
C3A—C4A—C5A—C6A	0.0 (6)	C5B—C6B—C7B—C8B	0.0 (6)
C4A—C5A—C6A—C7A	0.0 (6)	C6B—C7B—C8B—C3B	0.0 (5)
C4A—C5A—C6A—F1A	179.3 (4)	C14B—C9B—C10B—C11B	−0.7 (7)
F1A—C6A—C7A—C8A	−179.2 (3)	C15B—C9B—C10B—C11B	180.0 (4)
C5A—C6A—C7A—C8A	0.0 (6)	C10B—C9B—C14B—C13B	0.0 (8)
C6A—C7A—C8A—C3A	0.0 (5)	C15B—C9B—C14B—C13B	179.3 (5)
C15A—C9A—C14A—C13A	179.5 (3)	C10B—C9B—C15B—N2B	148.0 (4)
C10A—C9A—C15A—C1A	−150.2 (3)	C10B—C9B—C15B—C1B	−30.5 (6)
C14A—C9A—C15A—N2A	−148.0 (3)	C14B—C9B—C15B—N2B	−31.3 (6)
C10A—C9A—C15A—N2A	31.5 (4)	C14B—C9B—C15B—C1B	150.2 (4)
C14A—C9A—C10A—C11A	0.0 (5)	C9B—C10B—C11B—C12B	1.2 (7)
C15A—C9A—C10A—C11A	−179.5 (3)	C10B—C11B—C12B—F2B	179.0 (5)
C10A—C9A—C14A—C13A	0.0 (5)	C10B—C11B—C12B—C13B	−1.0 (9)
C14A—C9A—C15A—C1A	30.3 (4)	F2B—C12B—C13B—C14B	−179.7 (5)
C9A—C10A—C11A—C12A	0.0 (6)	C11B—C12B—C13B—C14B	0.3 (9)
C10A—C11A—C12A—F2A	179.4 (4)	C12B—C13B—C14B—C9B	0.2 (8)

### Hydrogen-bond geometry (Å, °)

**Table:**

Cg1, Cg4, Cg5 and Cg8 are the centroids of the C3A–C8A, C9C–C14C, C3B–C8B and C9D–C14D benzene rings, respectively.

**Table:**

*D*—H···*A*	*D*—H	H···*A*	*D*···*A*	*D*—H···*A*
O1A—H1A···N2B^i^	0.82	2.06	2.782 (4)	146
O1B—H1B···N2A	0.82	2.07	2.778 (4)	145
O2A—H2A···N1B	0.82	2.03	2.750 (4)	147
O2B—H2B···N1A^ii^	0.82	2.04	2.759 (4)	146
C10A—H10A···Cg5^iii^	0.93	2.94	3.690 (3)	139
C13A—H13A···Cg5^iv^	0.93	2.98	3.746 (3)	141
C14B—H14C···Cg1^iv^	0.93	2.94	3.702 (5)	140
C4C—H4CA···Cg8^iii^	0.93	2.92	3.674 (11)	139
C7C—H7CA···Cg8^iv^	0.93	2.97	3.749 (11)	143
C4D—H4DA···Cg4^iv^	0.93	2.90	3.651 (10)	139

Symmetry codes: (i) *x*, *y*+1, *z*; (ii) *x*, *y*−1, *z*; (iii) −*x*+1, −*y*+1, −*z*+1; (iv) −*x*, −*y*+1, −*z*+1.

## References

[bb1] Baktır, Z., Akkurt, M., Samshuddin, S., Narayana, B. & Yathirajan, H. S. (2011*a*). *Acta Cryst.* E**67**, o1262–o1263. 10.1107/S1600536811015455 PMC3089231 21754550

[bb2] Baktır, Z., Akkurt, M., Samshuddin, S., Narayana, B. & Yathirajan, H. S. (2011*b*). *Acta Cryst.* E**67**, o1292–o1293. 10.1107/S160053681101587X PMC3120319 21754699

[bb3] Bernstein, J., Davis, R. E., Shimoni, L. & Chang, N.-L. (1995). *Angew. Chem. Int. Ed. Engl.* **34**, 1555–1573.

[bb4] Farrugia, L. J. (1997). *J. Appl. Cryst.* **30**, 565.

[bb5] Farrugia, L. J. (1999). *J. Appl. Cryst.* **32**, 837–838.

[bb6] Fun, H.-K., Hemamalini, M., Samshuddin, S., Narayana, B. & Yathirajan, H. S. (2010*a*). *Acta Cryst.* E**66**, o582–o583. 10.1107/S1600536810004435 PMC2983722 21580348

[bb7] Fun, H.-K., Hemamalini, M., Samshuddin, S., Narayana, B. & Yathirajan, H. S. (2010*b*). *Acta Cryst.* E**66**, o864–o865. 10.1107/S1600536810009414 PMC2983895 21580687

[bb8] Jasinski, J. P., Guild, C. J., Samshuddin, S., Narayana, B. & Yathirajan, H. S. (2010*a*). *Acta Cryst.* E**66**, o2018. 10.1107/S1600536810026905 PMC3007578 21588329

[bb9] Jasinski, J. P., Guild, C. J., Samshuddin, S., Narayana, B. & Yathirajan, H. S. (2010*b*). *Acta Cryst.* E**66**, o1948–o1949. 10.1107/S1600536810026036 PMC3007318 21588274

[bb10] Oxford Diffraction (2007). *CrysAlis PRO* and *CrysAlis RED* Oxford Diffraction Ltd, Abingdon, England.

[bb11] Sheldrick, G. M. (2008). *Acta Cryst.* A**64**, 112–122. 10.1107/S0108767307043930 18156677

[bb12] Spek, A. L. (2009). *Acta Cryst.* D**65**, 148–155. 10.1107/S090744490804362X PMC2631630 19171970

